# Factors that influence patient and public adverse drug reaction reporting: a systematic review using the theoretical domains framework

**DOI:** 10.1007/s11096-023-01591-z

**Published:** 2023-05-29

**Authors:** Laila Shafei, Lina Mekki, Esraa Maklad, Turfa Alhathal, Rawan Ghanem, Rama Almalouf, Derek Stewart, Zachariah Nazar

**Affiliations:** College of Pharmacy, QU Health, Qatar University, Doha, Qatar

**Keywords:** Adverse drug reactions, Behaviour change, Direct patient reporting, Drug safety, Pharmacovigilance, Self-reporting, Theoretical domains framework, Under-reporting

## Abstract

**Background:**

Only 5–10% of all adverse drug reactions (ADRs) are reported. Mechanisms to support patient and public reporting offer numerous advantages to health care systems including increasing reporting rate. Theory-informed insights into the factors implicated in patient and public underreporting are likely to offer valuable opportunity for the development of effective reporting-interventions and optimization of existing systems.

**Aim:**

To collate, summarize and synthesize the reported behavioral determinants using the theoretical domains framework (TDF), that influence patient and public reporting of ADRs.

**Method:**

Cochrane, CINAHL, Web of science, EMBASE and PubMed were systematically searched on October 25th, 2021. Studies assessing the factors influencing public or patients reporting of ADRs were included. Full-text screening, data extraction and quality appraisal were performed independently by two authors. Extracted factors were mapped to TDF.

**Results:**

26 studies were included conducted in 14 countries across five continents. *Knowledge, social/professional role and identity, beliefs about consequences*, and *environmental context and resources,* appeared to be the most significant TDF domains that influenced patient and public behaviors regarding ADR reporting.

**Conclusion:**

Studies included in this review were deemed of low risk of bias and allowed for identification of key behavioural determinants, which may be mapped to evidence-based behavioral change strategies that facilitate intervention development to enhance rates of ADR reporting. Aligning strategies should focus on education, training and further involvement from regulatory bodies and government support to establish mechanisms, which facilitate feedback and follow-ups on submitted reports.

**Supplementary Information:**

The online version contains supplementary material available at 10.1007/s11096-023-01591-z.

## Impact statements

- Further focus on relevant theory is advocated to develop effective interventions to enhance the low rates of patient and public reporting of ADRs, and support medication safety.
- Patient and public reporting of adverse drug reactions are reported to be linked to their knowledge; their belief of the consequences of reporting; perceptions of their role in medications safety; and environmental context factors including the influence of healthcare professionals and the ease of reporting.
- Strategies to enhance patient and public ADR reporting should focus on education, training and further involvement from regulatory bodies and government support to establish mechanisms, which facilitate feedback and follow-ups on submitted reports.

## Introduction

Patient and public reporting of adverse drug reactions (ADRs) offers numerous advantages to health care systems, namely promotion of patient rights, earlier detection of important ADRs, and benefits to healthcare organizations from patient involvement [1, 2]. Similarly, there is growing evidence of their value in establishing stronger causality of ADRs [1-6]. Initially established to address substantial under-reporting rates of healthcare professionals, in recent years, systems to receive ADR reports from patients and public have become increasingly common globally [3, 5, 7]. The World Health Organization (WHO) Programme for International Drug Monitoring facilitates the exchange of information, policies, guidelines, and other normative activities between countries and support countries in their pharmacovigilance activities, including patient and public reporting [8]. However, there remains substantial under-reporting of ADRs by both healthcare professionals; and patients and public. It is estimated that only 5–10% of all ADRs are reported [9-18]. Under-reporting delays the triggering of signals and subsequently making decisions to maintain an appropriate drug benefit-to-harm balance [1].

Multiple articles, including recent investigations, have presented the factors contributing to healthcare professionals’ underreporting [10, 16, 19-24]. The findings have indicated that the knowledge and attitudes of healthcare professionals appear to be a significant influence; as well as a lack of time, different care priorities, uncertainty about the drug causing the ADR, difficulty in accessing reporting forms, lack of awareness of the requirements for reporting, and lack of understanding of the purpose of reporting systems. However, a review of these studies found only two that had adopted relevant theory to underpin the investigation; a study using the theory of planned behaviour to explain the mechanisms behind nurses intention to report ADRs [25]; and a study adopting the theory of satisfaction of needs to investigate predictive factors of reporting among community pharmacists [26].

Patient and public reporting of ADRs has also been investigated, although a large part of this research has focussed on the value of patient and public reporting to health systems [1, 6, 27, 28]. Such studies have included a 2017 systematic review [1], and numerous retrospective analyses evaluating the quality and characteristics of ADR reports by patients and public compared against those submitted by healthcare professionals [6, 27, 28].

Further studies [12, 29-33], including one systematic review [34], investigating the factors of patient and public reporting of ADRs have largely reported similar findings as those reported with healthcare professionals, and similarly, there has been little consideration of adopting theory to underpin these investigations.

A range of strategies that have attempted to address the high-rate of underreporting of ADRs in both healthcare professionals, and patients and public, have been reported in the literature [35, 36]. Two recent systematic reviews, one of which including a meta-analysis, reported on the effectiveness of interventions for improving ADR reporting by patients and public and healthcare professionals [35, 36]. Both reviews indicated that a range of strategies may be effective in increasing ADR reporting however, there is limited evidence of sustained improvement once the intervention ceases. Furthermore, Paudyal et al.’s review goes further to call for the need to develop and test theory-based interventions, since they point out that there is an accumulation of evidence that theory-based interventions are more likely to yield positive and sustainable results compared to pragmatic approaches [35]. These conclusions align with the UK Medical Research Council (MRC) guidance for the development and evaluation of complex interventions, which places emphasis on the importance of drawing on evidence and theory and specifying key steps in the intervention development process [37].

The Theoretical Domains Framework (TDF) [14] is one such theoretical framework that has that has been extensively and successfully adopted in healthcare practice research to investigate the various interacting components of specific behaviours. Beyond this, The Behavioural Change Wheel (BCW) builds upon the MRC guidance and provides a practical guide of how to develop theory and evidence based intervention [38]. The BCW is a systematic tool for designing complex interventions to understand behaviour(s), identify the theoretical process to facilitate behaviour change and specify intervention content [38]. Fundamental to the BCW is an appreciation that ‘Behaviour’ is influenced by an individual, or systems, ‘Capability, Opportunity and Motivation’ (COM-B model). The COM-B elements can further be mapped to theoretical constructs using the Theoretical Domains Framework (TDF) [39].

### Aim

The aim of this systematic review was to collate, summarize and synthesize the reported behavioural determinants using the TDF, that influence patient and public reporting of ADRs.

## Method

This systematic review was guided by the Preferred Reporting Items for Systematic Reviews and Meta-analysis (PRISMA) guidelines [40].

This systematic review protocol was pre-registered in Open Science Framework database (OPS) (Registration Number: 5J7WR) [41].

### Eligibility criteria and study selection

The review used the PICO framework to structure the eligibility criteria (Comparator was not relevant):

- Population: Adult (aged 16 years or over) patients, participants, and/or members of the public;
- Intervention: ADR reporting;
- Outcomes: Views, attitudes and/or knowledge.

All study designs were eligible for inclusion, except for reviews, letters, and editorials.

No date limitations were applied to the search; only studies published in English were included.

### Information sources and search strategy

An electronic database search was independently conducted by two authors on 25th October 2021. Five databases were systematically searched: PubMed, CENTRAL, EMBASE, Web of Science and CINAHL. Relevant medical subject headings (MeSH) and the detailed search strategies are provided in Supplementary File A. Reference lists of included studies were manually checked. The search included all articles published in English language since inception.

### Selection process

Pre-implementation studies, and studies which included pooled data from investigations that included other stakeholders (e.g. healthcare professionals) were excluded. Both title/abstract and full-text screening were completed by two independent authors and discrepancies were resolved by consulting a third author. All eligible articles were transferred to ENDNOTE 7 software for duplicates to be removed.

### Data collection process

A data extraction tool was developed using Excel software, extracting details of study design, country, year of publication, objective of the study, participants, description of data collection method, description of patient ADR reporting system, outcomes of the ADR reporting systems and the factors of patients and public ADR reporting.

### Risk of bias assessment

The Joanna Briggs Institute (JBI) checklists were used to critically appraise the cross-sectional and qualitative studies [42], while the Mixed Methods Appraisal Tool (MMAT) was used to appraise mixed methods studies [43]. The critical appraisal was independently conducted by two researchers, any disagreements were resolved by discussion with a third author.

### Data synthesis

The theoretical domains framework (TDF), a comprehensive framework derived from 33 psychological theories and 128 theoretical constructs and organized into 14 domains [14] was employed to classify the extracted data. Two researchers with experience using the TDF worked independently to classify the data into enablers and barriers (ZN and LS). To ensure consistent interpretation of the domains, continued reference was made to the original article describing the development of the TDF [14]. In assigning the data to the most relevant TDF domain, relevant contextual information reported for an individual barrier/enable were cross-referenced to TDF constructs to check for alignment.

Where disagreement arose, the two authors met to discuss. If the disagreement could not be resolved, a third experienced member of the research team (DS) was consulted, and discussions continued until consensus was reached. There were two main disagreements between the authors regarding the classification of two barriers/enablers into the most relevant TDF domains; one barrier that was aligning both to beliefs about consequence and goals; and one enabler that was aligning to both to intentions and goals.

## Results

### Search outcomes

**Fig. 1:**
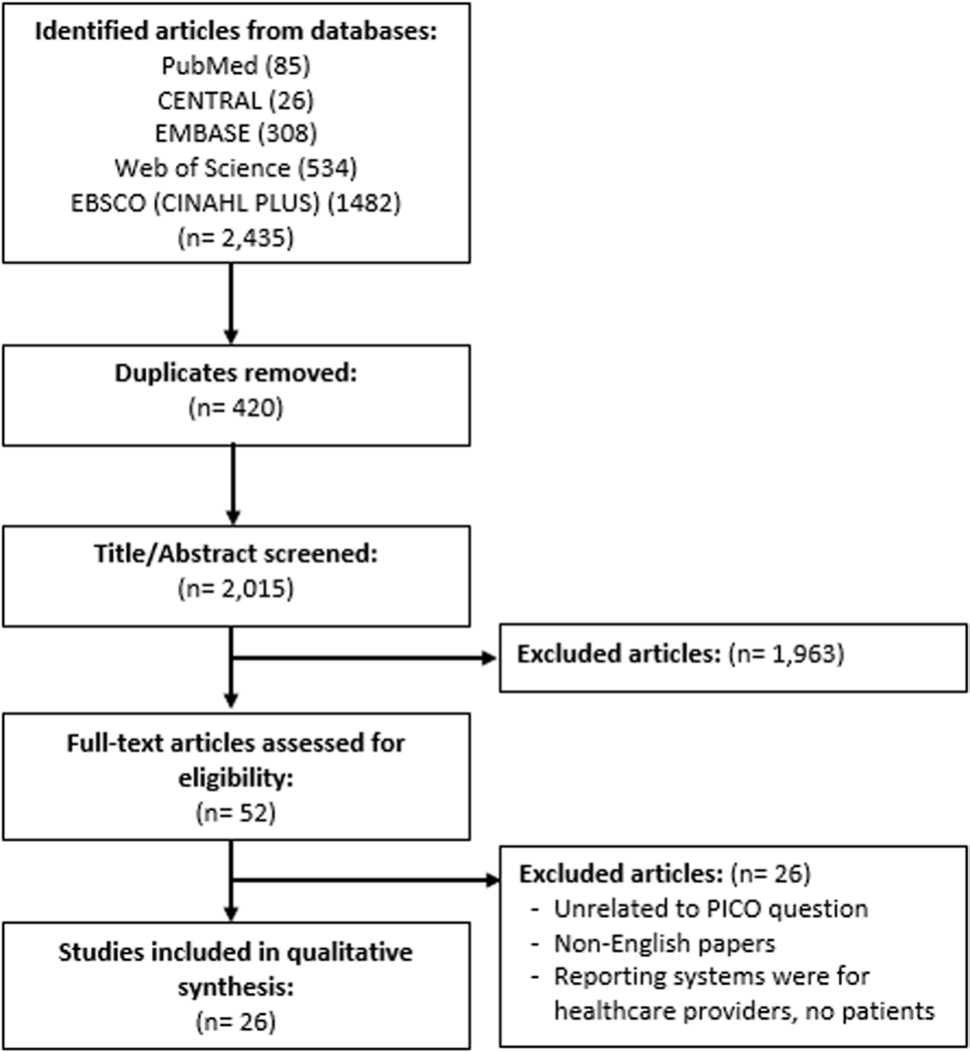
PRISMA flow diagram for selected articles

Twenty-six articles met the inclusion criteria [14, 29, 33, 44-66]. Figure 1 presents the PRISMA flow diagram. See electronic Supplementary File A, which provides further detail of study exclusion following full-text review. All studies were of cross-sectional design, adopting either qualitative (n = 7) or quantitative methods (n = 13); six articles adopted mixed methods. None of the studies had adopted relevant theories to support their investigations. Details of excluded studies with reasons of exclusion in electronic Supplementary File B.

### Study characteristics

Studies were published between 2008 and 2021, and were conducted in a range of countries; Saudi Arabia (n = 3) [49, 55, 57], United Kingdom (n = 4) [44, 58, 63, 64], USA (n = 2) [52, 53], Ghana (n = 3) [29, 48, 66] with remaining studies from South Korea [33], Netherlands [50, 62], Thailand [47], Canada [49], and Australia [14, 45, 59]. Detailed study characteristics are presented in Table 1.

**Table 1:** Description of included articles

Study	Study design	Country	Participants	Objectives
Anderson et al. [44]	Cross-sectional mixed methods	UK	1362 survey respondents and 27 interviewees, members of public who submitted a an ADR via Yellow Care Scheme (YCS)	To explore the opinions of patient reporters to the UK YCS
Adisa et al. [45]	Cross-sectional quantitative	Nigeria	1190 outpatients from various clinics at 3 public healthcare facilities in Ibadan	To evaluate patients’ knowledge, awareness, perception, and reporting of experienced ADRs
Ashoorian et al. [46]	Cross-sectional qualitative study	Australia	78 participants recruited through the Mental Health Commission	To assess the acceptability, content validity and usability of the My Medicines and Me (M3Q) self-report side effect questionnaire
Jarernsiripornkul et al. [47]	Cross-sectional mixed method	Thailand	2400 participants, at 3 locations: primary care units (PCU), community pharmacies and public areas	To determine confidence among members of the public in identifying suspected ADRs, information sources and their views towards direct ADR reporting
Sabblah et al. [48]	Cross-sectional quantitative	Ghana	442 Patients who had submitted ADR reports through the National Pharmacovigilance Centre	To explore feedback from patients regarding ADR reporting submitted to the National Pharmacovigilance Centre
Sales et al. [49]	Cross-sectional quantitative	Saudi Arabia	204 Members of the public	To assess the general public awareness and perception about ADRs reporting and pharmacovigilance
Sabblah et al. [29]	Cross-sectional quantitative	Ghana	442 patients from 2 community pharmacies in Ghana	To explore patients’ knowledge, attitudes, behaviors, and opinions on spontaneous ADR reporting in Ghana
Van Hunsel et al. [50]	Cross-sectional quantitative	Netherlands	1370 patients who had previously reported an ADR in Netherlands	To quantify the reasons and opinions of patients who reported ADRs to a pharmacovigilance center
Fortnum et al. [51]	Cross-sectional quantitative	UK	Not mentioned	To determine the awareness, previous experience in ADR reporting by the public in UK
McAuley et al. [52]	Cross-sectional quantitative	USA	82 Patients (or parents of patients) with epilepsy	To assess patients’ knowledge and attitudes toward reporting of ADRs secondary to anti-epileptic medicines
Oladimeji et al. [53]	Cross-sectional quantitative	USA	1220 internet surveys respondents selected from the Medicare database	To quantify the association between risk factors and beliefs about self-reported ADRs
Cheema et al. [54]	Cross-sectional quantitative	UK	256 patients visiting the emergency department of a UK hospital	To gain insight into patients’ medications and their experience of self-reported ADRs
Kim et al. [33]	Cross-sectional quantitative	South Korea	1000 adult participants across South Korea	To assess knowledge, attitude to report ADRs and explore the factors contributing to patients’ reporting
Kassem et al. [55]	Cross-sectional qualitative	Saudi Arabia	15 staff members from Unaizah College with experience with ADR reporting	To explore opinions and the need for patient friendly smartphone applications to enhance ADR reporting
Dweik et al. [56]	Cross-sectional qualitative	Canada	15 participants who had experienced ADRs	To explore patients’ experiences reporting ADRs and their views on usability of Canadian Vigilance reporting forms,
Islam et al. [57]	Cross-sectional qualitative study	Saudi Arabia	Patients who had used medicines in the last three months	To assess the public knowledge about medicine safety and ADR reporting
Arnott et al. [58]	Cross-sectional qualitative study	UK	44 parents with experience of submitting ADR report	To investigate parents’ views and experiences of direct reporting of a suspected ADR in their child
Braun et al. [59]	Cross-sectional quantitative	Australia	620 patients at 60 community pharmacies and rural pharmacies in three Australian states	To determine the prevalence of ADRs to over-the-counter complementary medicines and their severity by consumers; and to identify consumers’ reporting behaviors
Bukirwa et al. [60]	Cross-sectional qualitative study	Uganda	16 primary caregivers	To investigate perceptions and experience with existing pharmacovigilance systems
Elkalmi et al. [61]	Cross-sectional mixed method	Malaysia	334 members of general public in Penang	To explore the knowledge of the general population towards ADR and their reporting system
Harmark et al. [62]	Cross-sectional mixed method	Netherlands	21 patients participating in a web-based monitoring study of anti-diabetic drugs	To gain insight into patients’ motives for participating in active Post Marketing Surveillance and investigate their experiences with such a system
Kraska et al. [63]	Cross-sectional quantitative	UK	272 members of the public	To determine correlation between views of medicine safety, awareness of medicines’ side effects and reporting behaviors were related to experiences of suspected side effects
Lorimer et al. [64]	Cross-sectional qualitative	UK	15 patients admitted following a suspected ADR	To explore patients’ experiences of severe adverse reactions and their views on reporting their ADRs to YCS
Robertson and Newby [14]	Cross-sectional quantitative	Australia	2484 Members of the public	To determine levels of public awareness of consumer ADR reporting systems in Australia
Jha et al. [65]	Cross-sectional mixed method	Nepal	23 out-patients in a teaching hospital of Nepal	To assess the knowledge and perception towards pharmacovigilance among
Jacobs et al. [66]	Cross-sectional mixed methods	Ghana	572 patients from healthcare facilities	To assess patient awareness of ADRs and ADR-reporting and explores patient behaviors following an ADR

Four studies conducted in the UK investigated patients and public’ perceptions of the National Yellow Card Scheme (YCS) [44, 58, 63, 64]. Similarly, further studies, included investigations of national reporting systems and provided a description of the reporting system and the process for patient and public reporting, however, there were 7 studies in which such details were excluded or minimally reported [45, 47, 49, 54, 57, 59, 65].

There was also considerable diversity in the number of participants involved in the included studies, cross-sectional quantitative studies ranged between 84 and 2484 participants; qualitative studies ranged between 15 and 78 participants. Participants included in the studies were either members of the public, patients who had experienced ADRs previously (buy not necessarily reported it), or participants who were retrospectively approached following submission of their ADR through an ADR reporting system. Further detail of the ADR reporting systems and study participants are presented in electronic Supplementary File C.

### Risk of bias assessment

Risk of bias using the JBI checklist indicated that the influence of the researchers on the methodology in qualitative studies was not always reported [58, 64]. Within cross-sectional quantitative studies, reporting the assessment of confounding factors was frequently absent [48, 49, 54]. Assessment of mixed-methods studies using the MMAT tool revealed that the majority of studies were of high quality; however, one study did not perform sample size calculations, which drew some concerns regarding the external validity and generalizability of data [44]. Risk of bias results for both JBI and MMT tool represented in electronic Supplementary file D.

### Coding of factors influencing patients and public ADR reporting

A total of twelve TDF domains resonated from factors reported in the included articles. Examples of each domain are represented in Tables 2 and 3. The TDF domains memory, attention and decision processes and behavioral regulations did not seem to be implicated in patient and public reporting of ADRs.

**Table 2:** TDF domains: behavioral factors towards ADR reporting

TDF domain	Positive/negative influence to report ADR	Examples of factors and barriers extracted from the included articles
Knowledge	Positive	Participants were aware of the ADR reporting system used locally in their country [51]
	Negative	Patients believed that all drugs cause ADRs and assumed they would stop on cessation of therapy [44]
Skills	Positive	Patients with prior abilities and knowledge on how to report through the yellow card scheme were motivated to submit their ADR reports [57]
Social role/professional role and identity	Positive	Patients desired to have an active role in reporting ADRs [51] Others believed that it was their responsibility to prevent harm and support research. [49]
	Negative	Some patients indicated that it is not their job to report the ADRs [33]
Belief about capabilities	Positive	It was believed that reporting enhances consumers to attain self-efficacy of spontaneous reporting [33] Patients mentioned that reporting give them empowerment [45]
	Negative	Participants believed that they are not equipped to decide if an ADR had occurred [57]
Optimism	Positive	Participants wanted to help prevent others from experiencing similar ADRs [57] While others hoped to strengthen drug safety through reporting [64]
	Negative	Patients did not believe reporting will lead to improvements in the system and lacked optimism [33]
Belief about consequences	Positive	Some expressed that reporting is important to inform about the ADRs and their impact on lives [45] Reporting help in preventing harm to other people and contribute to drug safety [29]
	Negative	Some of the public do not report due to previous negative experiences after reporting such as neglect, underestimation, and denial [33]
Reinforcement	Positive	Having intolerable side effects impacting daily activities motivates patients to report [55] The need for additional medical care encourages individuals to report [59]
Intentions	Positive	Yellow card scheme offered patients welcome opportunity to voice their concerns about medicines that was not influenced by practitioners [53]
Goals	Positive	Patients report ADR because they aim to provide information about ADRs that are not in the patient information [49]
Environmental context and resources	Positive	Instructions provided by practitioners on self-reporting encourages patients to report [48]
	Negative	Some health practitioners’ attitudes do not take patients’ concerns about ADRs seriously [57]
Social influences	Positive	Patients were influenced by healthcare providers and family members to report their ADR [29]
	Negative	Patients do not report when pharmacists and doctors tell them about their side effects of their medications [60]
Emotion	Positive	Patients report because they have high concerns about ADRs [52]
	Negative	Some felt embarrassed to share their experiences with ADRs [50] Patients feel dissatisfied with how practitioners communicate ADRs [57]

**Table 3:** The distribution of TDF themes generated through included articles

Study	Anderson et al. (2011) [43]	Adisa et al. (2019) [44]	Ashoorian et al. (2015) [45]	Jarernsiripornkul et al. (2016) [46]	Sabblah et al. (2019) [47]	Sales et al. (2017) [48]	Sabblah et al. (2017) [29]	Van Hunsel et al. (2010) [49]	Fortnum et al. (2011) [50]	McAuley et al. (2009) [51]	Oladimeji et al. (2008) [52]	Cheema et al. (2018) [53]	Kim et al. (2020) [33]	Kassem et al. (2021) [54]	Dweik et al. (2020) [55]	Islam et al. (2020) [56]	Arnott et al. (2013) [57]	Braun et al. (2010) [58]	Bukirwa et al. (2008) [59]	Elkalmi et al. (2013) [60]	Harmark et al. (2013) [61]	Kraska et al. (2011) [62]	Lorimer et al. (2012) [63]	Robertson et al., (2013) [14]	Jha et al. (2014) [64]	Jacobs et al. (2018) [65]
Knowledge										**√**		**√**					**√**									**√**
Skills																	**√**									
Social/ professional role and identity								**√**		**√**			**√**			**√**	**√**			**√**	**√**					
Beliefs about capabilities			**√**										**√**				**√**									
Optimism	**√**						**√**						**√**				**√**									
Beliefs about consequences	**√**	**√**	**√**	**√**			**√**						**√**		**√**		**√**		**√**		**√**				**√**	**√**
Reinforcement															**√**				**√**			**√**				
Intentions																	**√**									
Goals							**√**	**√**																		
Environmental context and resources	**√**					**√**	**√**							**√**	**√**		**√**		**√**							
Social							**√**								**√**											
Emotion			**√**					**√**	**√**		**√**						**√**									

√: Domain was mapped to factors extracted from the study

*Knowledge*: Four studies reported factors that were mapped to *knowledge*; patients and public often lacked adequate understanding and knowledge about ADR self-reporting systems and the purpose of these systems. Studies were cross-sectional quantitative and qualitative that indicated patients and the public were aware of the available ADR reporting systems and the process for reporting, which subsequently encouraged reporting [52, 54, 58, 66].

*Skills*: One UK study preformed a cross-sectional qualitative methodology and concluded that patients who had acquired skills in reporting ADRs (either under the direction of a healthcare professional or an informed acquaintance) and had prior knowledge of how to use the Yellow Card Scheme seemed to be more motivated to submit ADR self-reports [58].

*Social /professional role and identity*: Seven studies reported factors influencing ADR reporting, as per our analysis, these factors were mapped to social/professional role and identity. Some studies indicated patients and the public belief that they themselves have an important role to report ADRs, and a responsibility to prevent harm and help support drug safety research [50, 52, 58, 62]. In contrast, four studies mentioned an opposing perception; patients and the public did not believe that ADR reporting is their responsibility, rather it is the healthcare providers’ duty [33, 57, 61, 62].

*Beliefs about capabilities*: There were conflicting reports regarding patients and the public belief in their ability to report ADRs; two studies reported positive beliefs [33, 46]; whereas, one study described that an important factor contributing to patient under-reporting was a self-perceived lack of necessary skill and inability to accurately identify ADRs [58].

*Optimism*: From four studies, our analysis mapped factors to optimism. Patients and the public were optimistic that their reports would help enhance drug safety and improve health systems [29, 44, 58]. Whereas one study indicated patients’ lack of optimism and belief that their reports would have no subsequent impact [33].

*Beliefs about consequences*: In six studies, participants indicated that they report ADRs because they recognize their importance in preventing harm and potential to positively impact on their lives [44-46, 62, 65, 66]. Additionally, patients and the public believed that their reports provide more information that contribute to improved medication safety [29, 47, 58, 66]. However, only three studies reported that patients and the public may avoid ADR reporting due to previous negative experiences such as dismissal, denial, and lack of feedback from the ADR reporting system [33, 56, 60].

*Reinforcements*: Three studies reported other stimuli that encouraged patient and public reporting, these included intolerable and severe drug reactions that impact daily living, and if patients and the public perceived the need for additional medical care to resolve the drug reaction [56, 60, 63].

*Intentions*: One study reported patients’ intention to report so to have a voice in sharing their concerns directly with the ADR reporting agency, without interference from healthcare professionals [58].

*Goals*: Two studies indicated that patient aim to provide information about ADRs that are not mentioned in the patient leaflets; therefore, they tend to report their ADRs whenever it occurs [29, 50].

*Environmental context and resources*: Three studies mentioned that when patients and the public received specific directions and reminders from healthcare providers, they were more motivated to report their suspected ADR [49, 55, 58]. Based on the analysis, these factors were mapped to environmental context and resources. However, practitioners’ dismissive attitudes; the cost of accessing the ADR reporting system; were environmental factors that discouraged patients and the public from reporting [44, 58, 60]. Additionally, one study mentioned that having an ADR system that is easily and freely accessible and not limited to clinic-opening times was preferred [55]. Participants across various investigations agreed that a system that was easily and freely accessible, that was not limited to clinic-opening times was preferred [47, 48, 54]. Two studies reported that the use of technology such as mobile phones or web-based platforms were more preferred than paper-reports with an integrated function to provide feedback following submissions of reports [47, 48].

*Social influences*: Patient self-reporting was encouraged by family members and healthcare providers [29, 56].

*Emotion*: Two studies revealed that patients and the public anxiety resulting from suffering an ADRs is what encouraged them to report [50, 53]. Moreover, patients and the public annoyance and dissatisfaction with practitioners’ response; motivated them to report [46, 58]. In contrast, one article mentioned that patients indicated feeling embarrassed to report specific ADRs may have prevented them from reporting [51].

## Discussion

### Key findings

This systematic review has elucidated the key behavioral determinants relating to patient reporting of ADRs as reported in the literature. Under-reporting by patients and the public of ADRs is well documented, this review identified factors that aligned to 12 of the 14 TDF domains. *Social role and identity; beliefs about consequences; knowledge; environment context and resources*, were the domains that were most frequently identified to either positively or negatively influence reporting.

There were no noticeable contrasting trends in findings reported from the various countries or settings; similarly both findings derived from qualitative, quantitative or mixed-methods studies were not distinctly dissimilar.

Most studies were found to be of high quality and low-risk of bias. However, this finding should be considered in consideration of the heterogeneity between various tools that were used. The JBI checklist [41] offers a more comprehensive set of criteria than the MMAT; thus as well as the MMAT increasing the need for interpretation, its cumulative critical appraisal provides a less detailed evaluation.

### Strengths and weaknesses

This review is the first to adopt behavioural theory to investigate and synthesize reported influencers of patient and public reporting of ADRs. These findings facilitate elucidation of key issues to be considered in refining existing structures and processes and increase the effectiveness of patient and public reporting ADR systems.

In terms of the study limitations, the search was limited to studies published in English or where an English translation was available. Secondly, the review excluded studies that investigated the factors of ADR reporting in population samples that included both patients and the public, and healthcare professionals with pooled findings, where it was not possible to distinguish the specific factors of each of these populations. It should be mentioned, however, that such studies were few and included small sample sizes.

### Interpretation

This review builds on the existing knowledge base and provides deeper insights into the issues regarding patient and public reporting ADRs. Whilst some of the findings, such as: participant’s confusion as to who’s responsibility it is to report ADRs and to whom; patients and the public belief that reporting may prevent similar ADRs in others; that reporting would subsequently improve drug safety; and that reporting may improve the practice of health care; are similar to those reported in previous studies [29-33, 56]. This study has provided rich description regarding the TDF domains which influence reporting.

Many of the included studies reported patient and public uncertainty regarding reporting, which was mapped to the TDF domains *knowledge* and *social role and identity*. Further studies are warranted to understand why patients and the public experience this uncertainty, and to investigate a possible link to reports of healthcare professionals’ sub-optimal knowledge, attitudes, time constraints and lack of motivation regarding ADR reporting [9-11, 67, 68]. Recent literature has advocated that healthcare providers have a responsibility to enhance patients and the public awareness and understanding of their role through various methods such as sending them reminders and face-to-face educational sessions [35]. Furthermore, multiple studies reported patients and the public belief that their reports would contribute to subsequent improvements in drug safety and/or health care delivery, which was mapped to the TDF domain *beliefs about consequences.* An akin approach that has proved successful in encouraging healthcare professionals to report ADRs is dissemination by email of submitted reports, such investigations have not been conducted with patients and the public [69-71].

The TDF domain *environment context and resources*, captured the various reports which emphasized the patients and the public preference for a reporting system that is cost-free, easily accessible and confidential. A digital platform was also proposed several times by study participants. Such characteristics are in-line with WHO guidelines that suggests that reporting for general public should be easy and cheap [72]. Moreover, Leonardi’s Methodological Guidelines for the Study of Materiality and Affordances, discusses the need to recognise such characteristics (and others) of digital technologies in order to engage users and promote usability [73].

Further interventions aimed at improving ADR reporting rates have been reported in a 2020 systematic review and meta-analysis, which revealed that of the 28 included studies, the vast majority were educational interventions aimed at healthcare professionals [35]. The review commented on the lack of high-quality theory-informed interventions reported in the literature and their limited evidence of enhancing rates of reporting. Additionally, the authors emphasise the need to develop theory-based interventions, which are more likely to yield positive and sustainable results compared to pragmatic approaches.

This review has benefitted from the use of TDF to identify key behavioural domains that can be used to target in developing and/or refining patient and public reporting systems, as suggested by the UK Medical Research Council [37]. The Behaviour Change Wheel (BCW) advises which domains promote optimal strategies and designing of interventions mapped to behavioural determinants. Interventions are described as seven categories of education; persuasion; incentivisation; coercion; training; restriction; and environmental restructuring, modelling and enablement [38]. Determinants of *social role and identity* and *beliefs about consequences* are linked to *reflective motivation* in the BCW and may be enhanced through education, enablement and training. Similarly, the TDF determinant of *knowledge* is linked to *psychological capability* and may be enhanced through an educational intervention. *Environmental context and resources* is linked *physical opportunities* of the BCW may be enhanced through training, environmental restructuring and enablement [37, 38].

Thus, education, enablement and training are the most relevant interventions to influence reporting behaviours and enhance reporting-rates. This may be achieved through establishing accessible step-by-step instructional protocols and educational resources for patient and public reporting; promoting reporting through national awareness campaigns including education about the benefits of self-reporting; and integration into the delivery of person-centred care to enhance the advocacy from healthcare professionals [37, 38].

Also environmental restructuring which is likely to include further involvement from regulatory bodies and government support to establish mechanisms, which facilitate feedback and follow-ups on submitted reports, and implemented with the necessary modifications to regulations, legislations, and service provision [37, 38].

### Further research

In light of these findings, this review may be used as a guide in future attempts to develop and refine patient and public ADR reporting system. Carefully designed interventions that further patient and public engagement; and enhance stakeholder education, awareness and attitudes towards ADR reporting, are likely to result in more increased reporting rates. Accordingly, it has been reported that countries with the highest reporting rates were found to intensively engage with consumer organisations to promote awareness and acceptance of pharmacovigilance [50]. This study also indicates the structural characteristics which may support patient and public reporting; simple and convenient access through a web-enabled platform or mobile application seem to support reporting rates. Lenoardi’s methodological guidelines has shown potential alignment with these findings to account for materiality and affordances of the reporting system [51] and may be adopted in future studies to elucidate further value.

Lastly, this study is the first to adopt the TDF to understand patients and the public behaviour towards reporting, albeit using retrospective data; future prospective studies, underpinned with behavioural theory, are also of value to corroborate findings and provide further insight. Likewise, the authors advocate for interventional studies, informed by the emerging evidence-based, and underpinned with relevant theory, to assist in developing strategies to optimise patient and public reporting.

## Conclusion

This review has revealed the key behavioural determinants that influence patient and public reporting ADRs; namely, their knowledge; their sense of their social role and identity; their beliefs about the consequences of reporting; and their environmental context. These determinants can be mapped to behavioural change strategies to facilitate intervention development and enhance rates of ADR reporting.

## Supplementary Information

Below is the link to the electronic supplementary material.

Supplementary file1 (DOCX 57 kb)
