# Supplementary material for: Factors that influence patient and public adverse drug reaction reporting: a systematic review using the theoretical domains framework
Source: Int J Clin Pharm. 2023 May 29;45(4):801–13. doi: 10.1007/s11096-023-01591-z (PMC10366238; doi:10.1007/s11096-023-01591-z)
Supplement: Supplementary file 1 — Supplementary file1 (DOCX 57 kb) [file 11096_2023_1591_MOESM1_ESM.docx]

**Electronic Supplementary Material:**

**Electronic Supplementary Material 1: Search strategies adopted in each database**

| **Database** | **Search Strategy** |
| --- | --- |
| **PubMed** | (((("Patients"[Mesh]) AND "Adverse Drug Reaction Reporting Systems"[Mesh])) OR "Drug-Related Side Effects and Adverse Reactions"[Mesh]) AND "Pharmacovigilance"[Mesh] |
| **EMBASE** | 'patient'/exp AND 'self report'/exp AND ('adverse drug reaction'/exp OR 'pharmacovigilance'/exp) |
| **CENTRAL** | Patient [Mesh] AND (self-report[Mesh]OR ADR reporting[Mesh] ) AND ADR[Mesh] AND Pharmacovigilance [Mesh] |
| **Web of Science** | ((*patient* OR public) AND (*self report* OR *self-report*) AND (*Adverse drug reaction* OR *pharmacovigilance*)) |
| **EBSCO (CINAHL PLUS)** | Patient AND report AND “ADR” (IN ABSTRACT) |

**Electronic Supplementary Material 2: Reasons of exclusion table**

| **Author** | **Year** | **Title** | **Reason** |
| --- | --- | --- | --- |
| Dweik et al | 2016 | Factors affecting patient reporting of adverse  drug reactions: a systematic review | Systematic review |
| Chaipichit et al | 2015 | Introducing self-reporting questionnaires about adverse drug reactions to patients: Attitude towards their first experience | Conference paper |
| Vries et al | 2019 | Use of a Patient‐Friendly Terms List in the Adverse Drug Reaction Report Form: A Database Study | Database study |
| Gonzalez et al | 2013 | Strategies to Improve Adverse Drug Reaction Reporting: A Critical and Systematic Review | Systematic review |
| Holch et al | 2016 | Asking the right questions to get the right answers: using cognitive interviews to review the acceptability, comprehension and clinical meaningfulness of patient self-report adverse event items in oncology patients | Study is not reporting factor or influencers of patient ADR reporting |
| Pusturee et al | 2015 | Survey of patients’ experiences and their certainty of suspected adverse drug reactions | Study is not reporting factor or influencers of patient ADR reporting |
| Olver et al | 2018 | The timeliness of patients reporting the side effects of chemotherapy | Study is not reporting factor or influencers of patient ADR reporting |
| Ploen et al | 2015 | User-Driven Development of a Web-Based Tool for Patient Reporting of Drug-Related Harm | Study is not reporting factor or influencers of patient ADR reporting |
| Ribeiro-Vaz et al | 2016 | How to promote adverse drug reaction reports using information systems – a systematic review and meta-analysis | Systematic review and not related to our PICO question |
| Jarensiripornkul et al | 2012 | Patient reporting of suspected adverse drug reactions to antiepileptic drugs: Factors affecting attribution accuracy | Study is not reporting factor or influencers of patient ADR reporting |
| Shiyanbola et al | 2010 | Concerns and Beliefs About Medicines and Inappropriate Medications: An Internet-Based Survey on Risk Factors for Self-Reported Adverse Drug Events Among Older Adults | Assessing self-report of adverse drug events including allergic reaction due to medications which is not our scope |
| Florence et al | 2010 | What Motivates Patients to Report an Adverse Drug Reaction? | Letter |
| Riodran et al | 2020 | Stakeholders’ knowledge, attitudes and practices  to pharmacovigilance and adverse drug reaction reporting in clinical trials: a mixed methods study | ADR reporting is assessed among stakeholders not patients |
| Valinciute-jankauskiene et al | 2021 | Adverse Drug Reaction Reporting by Patients in 12 European Countries | Study is not reporting factor or influencers of patient ADR reporting |
| Assanee et al | 2021 | Factors influencing patient intention to report adverse drug reaction to community pharmacists: A structural equation modeling approach | ADR reporting is assessed among healthcare providers not patients |
| Januskiene et al | 2020 | What are the patients' and health care professionals’  understanding and behaviours towards adverse drug reaction reporting and additional monitoring? | Did not specify whether the ADR reporting influencers mentioned by healthcare providers or patients |
| Fracas et al | 2010 | Patients reporting of suspected adverse reactions to antidepressants. A pilot methodological study | Study is developing method for patients reporting, not assessing influencers of reporting |
| Gujral et al | 2010 | Public awareness of the yellow card scheme for reporting ADRs | Systematic review |
| Lebanova et al | 2014 | Study of patients’ potential as a source for spontaneous reporting systems in Bulgaria | Non-English study |
| Matos et al | 2014 | Are patients ready to take part in the pharmacovigilance system: a Portuguese preliminary study concerning drug reaction reporting. | Conference paper |
| Parrella et al | 2014 | Consumer reporting of adverse events following immunization (AEFI): identifying predictors of reporting an AEFI. | Study is not reporting factor or influencers of patient ADR reporting |
| Qamar et al | 2014 | Reporting of adverse drug reactions by the end users-patients in Pakistan | Non-English study |
| Rofles et al | 2014 | Important information regarding reporting of adverse drug reactions: a qualitative study | Study is not reporting factor or influencers of patient ADR reporting |
| Salvo et al | 2013 | Attitudes and opinion about adverse drug events of women living in a city of south Italy. | Study is not reporting factor or influencers of patient ADR reporting |
| Hunsel et al | 2010 | What motivates patients to report an adverse drug reaction? | Letter |

| **Electronic Supplementary Material 3: Description of studies methodologies and ADR reporting systems** | | |
| --- | --- | --- |
| **Study** | **Description of data collection method** | **Description of patient-reporting ADR system used** |
| **Anderson et al. (2011) [43]** | Questionnaires and Semi-structured telephone interviews. | Yellow Card Scheme (YCS) operated by the UK Medicines and Healthcare products Regulatory Agency |
| **Adisa et al. (2019) [44]** | Semi-structured questionnaire | N/A |
| **Ashoorian et al. (2015) [45]** | Focus groups | My Medicines and Me Questionnaire (M3Q) is a questionnaire for identifying medication side effects that mental health patients are experiencing and their perceptions of these  effects |
| **Jarernsiripornkul et al. (2016) [46]** | Self-administered questionnaire and Semi-structured interviews | N/A |
| **Sabblah et al. (2019) [47]** | Questionnaire and face-to-face interview | Patients submit ADR reports to National pharmacovigilance system (NPvC) in Ghana |
| **Sales et al. (2017) [48]** | Validated self-administered questionnaire | N/A |
| **Sabblah et al. (2017) [29]** | Questionnaire and face-to-face interviews | National Pharmacovigilance Centre (NPvC) in Ghana developed a blue form intended for patient-reporting ADRs. The form contains information on reporter details, details of the individuals who experienced the ADR, the ADR details and the suspected product details. |
| **Van Hunsel et al. (2010) [49]** | Questionnaire | Electronic reporting form on the Lareb website; the preferred reporting method by the pharmacovigilance center |
| **Fortnum et al. (2011) [50]** | Telephone omnibus survey | Yellow Card Scheme |
| **McAuley et al. (2009) [51]** | Questionnaire | MedWatch (FDA Safety Information and Adverse Event Reporting Program). It has a web-based reporting system and paper-based reporting system. |
| **Oladimeji et al. (2008) [52]** | Internet survey | Self-reporting is seeing a doctor/ pharmacist about any unwanted reaction from their medication within one year prior to recruitment. |
| **Cheema et al. (2018) [53]** | Questionnaire | N/A |
| **Kim et al. (2020) [33]** | Questionnaire | ADRs are reported in a form named ‘Individual Case Safety Reports (ICSRs) which can be submitted by the public, HCPs, manufacturing companies via call center, fax or email to the pharmacovigilance system of South Korea |
| **Kassem et al. (2021) [54]** | Semi-structured interview | SFDA Reporting System. A patient ADR reporting form (online) was developed by the Saudi FDA several years ago |
| **Dweik et al. (2020) [55]** | An in-person, telephone, or Skype interview | Patients can report ADRs directly to Health Canada through an electronic reporting form, telephone, or paper form using MedEffect website |
| **Islam et al. (2020) [56]** | Questionnaire | N/A |
| **Arnott et al. (2013) [57]** | Semi-structured telephone and face-to-face interviews | Yellow Card Scheme |
| **Braun et al. (2010) [58]** | Self-administered questionnaire | N/A |
| **Bukirwa et al. (2008) [59]** | Focus group discussions | National Drug Authority (NDA) introduced a standardized report form into the public sector |
| **Elkalmi et al. (2013) [60]** | Face-to-face interview | Reports submitted to Malaysian Adverse Drug Reactions ADR Advisory  Committee (MADRAC) |
| **Harmark et al. (2013) [61]** | Face-to-face interview | Lareb  Intensive Monitoring (LIM) system |
| **Kraska et al. (2011) [62]** | Face-to-face interview | Yellow card scheme |
| **Lorimer et al. (2012) [63]** | Face-to-face interview | Yellow card scheme |
| **Robertson & Newby. (2013) [14]** | Telephone interviews and online surveys | Direct reports to Therapeutic Goods Administration (TGA) |
| **Jha et al. (2014) [64]** | Face-to-face interview | N/A |
| **Jacobs et al. (2018) [65]** | Surveys followed by face-to-face interviews | Ghana-FDA Patient Reporting System |

**Electronic Supplementary Material 4: Risk of bias assessment results**

**JBI risk of bias assessment for cross-sectional and qualitative studies:**

| Score based on appropriate JBI appraisal* | | | | | | | | | | | | |
| --- | --- | --- | --- | --- | --- | --- | --- | --- | --- | --- | --- | --- |
| Study | **Design** | **1** | **2** | **3** | **4** | **5** | **6** | **7** | **8** | **9** | **10** |  |
| Adisa et al. (2019) [45] | Qualitative | √ | √ | √ | √ | √ | √ | ? | √ | √ | √ |  |
| Ashoorian et al. (2015) [46] | Qualitative | √ | √ | √ | √ | √ | √ | ? | ? | √ | √ |  |
| Sabblah et al. (2019) [48] | Cross-sectional | √ | √ | √ | √ | X | X | √ | √ | NA | NA |  |
| Sales et al. (2017) [49] | Cross-sectional | X | √ | √ | √ | √ | X | √ | √ | NA | NA |  |
| Sabblah et al. (2017) [29] | Cross-sectional | √ | √ | √ | √ | √ | √ | √ | √ | NA | NA |  |
| Van Hunsel et al. (2010) [50] | Cross-sectional | √ | √ | √ | √ | ? | ? | √ | √ | NA | NA |  |
| Fortnum et al. (2011) [51] | Cross-sectional | √ | √ | √ | √ | √ | √ | √ | √ | NA | NA |  |
| McAuley et al. (2009) [52] | Cross-sectional | X | √ | √ | √ | √ | √ | √ | √ | NA | NA |  |
| Oladimeji et al. (2008) [53] | Cross-sectional | √ | √ | √ | √ | X | X | √ | √ | NA | NA |  |
| Cheema et al. (2018) [54] | Cross-sectional | √ | √ | √ | √ | X | √ | √ | √ | NA | NA |  |
| Kim et al. (2020) [33] | Cross-sectional | √ | √ | √ | √ | X | √ | √ | √ | NA | NA |  |
| Kassem et al. (2021) [55] | Qualitative | √ | √ | √ | √ | √ | √ | √ | √ | √ | √ |  |
| Dweik et al. (2020) [56] | Qualitative | √ | √ | √ | √ | √ | √ | √ | √ | √ | √ |  |
| Islam et al. (2020) [57] | Cross-sectional | √ | √ | √ | √ | X | ? | √ | √ | NA | NA |  |
| Arnott et al. (2013) [58] | Qualitative | √ | √ | √ | √ | √ | ? | ? | √ | X | √ |  |
| Braun et al. (2010) [59] | Cross-sectional | X | √ | √ | √ | X | X | √ | ? | NA | NA |  |
| Bukirwa et al. (2008) [60] | Qualitative | √ | √ | √ | √ | √ | X | √ | √ | X | √ |  |
| Kraska et al. (2011) [63] | Cross-sectional | X | ? | √ | √ | ? | ? | √ | ? | NA | NA |  |
| Lorimer et al. (2012) [64] | Cross-sectional | X | X | √ | √ | ? | ? | √ | √ | NA | NA |  |
| Robertson & Newby. (2013) [14] | Qualitative | √ | √ | √ | √ | √ | X | X | √ | √ | √ |  |
| *Appropriate appraisal for either cross-sectional or qualitative studies was used.  Cross-sectional: 8 criteria; qualitative: 10 criteria  √= yes, X= No, ?= unclear, NA=not applicable  Quality appraisal using MMAT tool for mixed method studies   \| MMAT methodological quality criteria \| \| \| \| \| \| \| \| \| \| \| \| \| \| \| \| \| --- \| --- \| --- \| --- \| --- \| --- \| --- \| --- \| --- \| --- \| --- \| --- \| --- \| --- \| --- \| --- \| \| Study \| **Section I: Qualitative** \| \| \| \| \| **Section II: Quantitative descriptive** \| \| \| \| \| **Section III: Mixed methods** \| \| \| \| \| \|  \| **1.1** \| **1.2** \| **1.3** \| **1.4** \| **1.5** \| **4.1** \| **4.2** \| **4.3** \| **4.4** \| **4.5** \| **5.1** \| **5.2** \| **5.3** \| **5.4** \| **5.5** \| \| Anderson et al. (2011) [44] \| √ \| √ \| √ \| √ \| √ \| √ \| X \| X \| √ \| √ \| √ \| √ \| √ \| √ \| √ \| \| Jarernsiripornkul et al. (2016) [47] \| √ \| √ \| √ \| √ \| √ \| √ \| √ \| √ \| √ \| √ \| √ \| √ \| √ \| √ \| √ \| \| Elkalmi et al. (2013) [61] \| √ \| √ \| √ \| √ \| √ \| √ \| √ \| √ \| √ \| √ \| √ \| √ \| √ \| √ \| √ \| \| Harmark et al. (2013) [62] \| √ \| √ \| √ \| √ \| √ \| √ \| √ \| √ \| √ \| √ \| √ \| √ \| √ \| √ \| √ \| \| Jha et al. (2014) [65] \| √ \| √ \| √ \| √ \| √ \| √ \| √ \| √ \| √ \| √ \| √ \| √ \| √ \| √ \| √ \| \| Jacobs et al (2018)[66] \| √ \| √ \| √ \| √ \| √ \| √ \| √ \| √ \| √ \| √ \| √ \| √ \| √ \| √ \| √ \| \| √=yes,X=No,?=unclear \| \| \| \| \| \| \| \| \| \| \| \| \| \| \| \| | | | | | | | | | | | | |
